# Integrated Echo-Hemodynamic Framework for VA-ECMO in Right Ventricular Infarction

**DOI:** 10.1016/j.jaccas.2026.107617

**Published:** 2026-03-25

**Authors:** Shigeki Horiguchi, Makoto Hirose, Tetsuya Tobaru, Hiroyasu Uzui, Hiroshi Tada

**Affiliations:** a Department of Cardiovascular Medicine, Chiba-Nishi General Hospital, Matsudo, Japan; b Department of Cardiovascular Medicine, University of Fukui Hospital, Fukui, Japan

**Keywords:** cardiogenic shock, mechanical circulatory support, nitric oxide, pulmonary artery pulsatility index, RV-PA coupling, weaning

## Abstract

Right ventricular (RV) myocardial infarction with cardiogenic shock presents complex management challenges. We present 3 cases illustrating a potential integrated approach combining pulmonary artery pulsatility index (PAPI) and RV-pulmonary artery coupling ratios (tricuspid annular systolic velocity/pulmonary artery systolic pressure, tricuspid annular plane systolic excursion/pulmonary artery systolic pressure) for venoarterial extracorporeal membrane oxygenation decision-making. Case 1 (PAPI = 0.47) and case 2 (PAPI = 0.64) required venoarterial extracorporeal membrane oxygenation with successful weaning when PAPI exceeded 1.5. Case 3 (PAPI = 1.05) was managed conservatively. In case 2, inhaled nitric oxide (iNO) improved PAPI during weaning. In case 3, iNO caused paradoxical deterioration necessitating discontinuation. Based on these observations, we suggest that PAPI and coupling ratios provide complementary information: PAPI reflects hemodynamic reserve, whereas coupling ratios assess intrinsic RV function. Neither parameter alone is sufficient; integration of both guides clinical decisions. Prospective validation is needed. For iNO use without mechanical support, adequate left ventricular reserve is critical; PAPI decline mandates immediate discontinuation.

## Visual Summary

**Figure:**
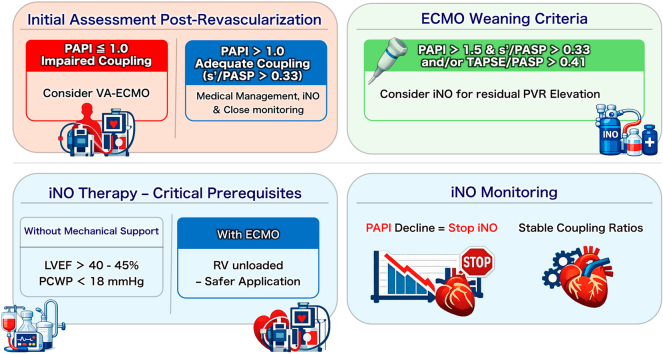

Right ventricular myocardial infarction (RVMI) complicates 10% to 15% of ST-segment elevation myocardial infarctions, representing a critical subset with distinct hemodynamic challenges and increased mortality.^1^ The pathophysiology involves acute ischemia of the afterload-sensitive right ventricle (RV), resulting in elevated right atrial pressure, reduced RV stroke volume, and diminished left ventricular (LV) preload through ventricular interdependence, precipitating cardiogenic shock in up to 40% of cases.^2^^,^^3^ When medical therapy fails, venoarterial extracorporeal membrane oxygenation (VA-ECMO) provides essential circulatory support.

**Take-Home Messages**

• Unlike load-dependent parameters such as right ventricular fractional area change, afterload-normalized echocardiographic coupling ratios (specifically tricuspid annular systolic velocity/PASP >0.33 and tricuspid annular plane systolic excursion/PASP >0.41) may reliably reflect intrinsic right ventricular recovery during VA-ECMO despite mechanical unloading.
• We propose an integrated framework where these echocardiographic markers, combined with hemodynamic pulsatility (pulmonary artery pulsatility index >1.5), guide critical decisions regarding ECMO initiation and successful weaning.
• Furthermore, echocardiographic assessment of left ventricular reserve is essential before inhaled nitric oxide therapy to identify patients at risk of paradoxical decompensation due to increased venous return.

However, VA-ECMO dramatically unloads the right heart by diverting venous return, artificially improving conventional echocardiographic parameters such as tricuspid annular plane systolic excursion (TAPSE) and RV fractional area change (RVFAC). This mechanical unloading masks persistent dysfunction and can lead to premature weaning failure.^4^^,^^5^ The pulmonary artery pulsatility index (PAPI), calculated as: (pulmonary artery [PA] systolic pressure [PASP] − PA diastolic pressure)/central venous pressure (CVP), reflects RV hemodynamic reserve and has emerged as a valuable tool in RV failure assessment.^6^ Unlike load-dependent echocardiographic parameters, PAPI integrates RV stroke work with filling pressure.

**Figure 1:**
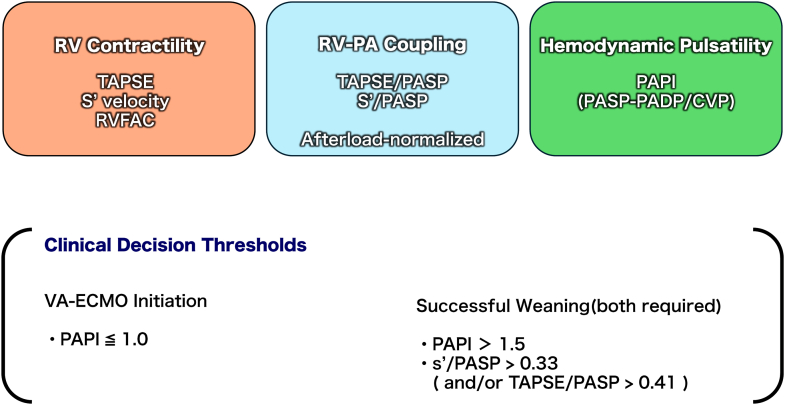
Clinical Decision Thresholds and Definitions Overview of the hemodynamic and echocardiographic parameters used in the proposed integrated framework. (Top) Definitions of key parameters: right ventricular (RV) contractility markers (TAPSE, s', and RVFAC), afterload-normalized RV-pulmonary artery coupling ratios (TAPSE/PASP and s'/PASP), and the hemodynamic pulsatility index (PAPI). (Bottom) Proposed clinical decision thresholds based on the integration of these parameters. A PAPI ≤ 1.0 suggests severe RV failure potentially requiring VA-ECMO. For successful weaning, a PAPI >1.5 combined with preserved coupling (s'/PASP >0.33 and/or TAPSE/PASP >0.41) is required to ensure intrinsic RV recovery. CVP = central venous pressure; PADP = pulmonary artery diastolic pressure; PAPI = pulmonary artery pulsatility index; PASP = pulmonary artery systolic pressure; RVFAC = right ventricular fractional area change; s' = tricuspid annular systolic velocity; TAPSE = tricuspid annular plane systolic excursion; VA-ECMO = venoarterial extracorporeal membrane oxygenation.

RV-PA coupling ratios (tricuspid annular systolic velocity [s']/PASP, TAPSE/PASP) offer afterload-normalized assessment of RV function, approximating the relationship between end-systolic elastance and arterial elastance.^7^ Recent evidence from Park et al^8^ demonstrated that s'/PASP >0.33 was an independent predictor of successful ECMO weaning (sensitivity = 89%, specificity = 92%), with TAPSE/PASP >0.41 also showing a predictive value. These findings inform our integrated “echo-hemodynamic triad” approach combining RV contractility measures, afterload-normalized RV-PA coupling ratios (with emphasis on s'/PASP as the primary predictor), and hemodynamic pulsatility (PAPI) for comprehensive VA-ECMO management in RVMI (Figure 1).

## Case Presentations

### Case 1: Delayed presentation with failed initial weaning

A 69-year-old woman with hypertension, diabetes, dyslipidemia, and active smoking presented with delayed inferior ST-segment elevation (Table 1) and complete atrioventricular block. Her blood pressure was 144/82 mm Hg on dopamine and heart rate was 38 beats/min, with jugular venous distention, bilateral rales, and diaphoresis. Laboratory findings revealed acute infarction (creatine kinase level = 1,183 U/L, troponin level = 118,993 pg/mL) and end-organ damage (aspartate aminotransferase level = 486 U/L, creatinine level = 1.73 mg/dL). Echocardiography showed a severely dilated akinetic RV with a small LV cavity, suggesting ventricular interdependence. Emergency angiography revealed total proximal right coronary artery (RCA) occlusion (RCA #2); percutaneous coronary intervention (PCI) achieved only TIMI flow grade 2 due to thrombus burden.

**Table 1:** Patient Characteristics and Initial Presentation

	Case 1	Case 2	Case 3
Age, y/sex	69/F	68/M	83/F
Risk factors	HTN, DM, DLP, smoking	DM, smoking, CKD	HTN
Culprit vessel	RCA #2	RCA #2 (in-stent)	LCx #11 (supplies RV; RCA hypoplasia)
PCI result	TIMI flow grade 2	TIMI flow grade 3	TIMI flow grade 3
Initial lactate (mmol/L)	7.1	7.1	5.2
SCAI class	C to D-E	C to D-E	C
Initial PAPI	0.47	0.64	1.05
Baseline PCWP (mm Hg)	N/A	N/A	12-14
Echo	LV inferior mild hypokinesis, RV akinesis, LVEF 58.7%	LV inferior mild hypokinesis, RV akinesis, LVEF 56.9%	LV posterior-inferior severe hypokinesis, RV akinesis, LVEF 39.0%
MCS decision	ECMO initiated	ECMO initiated	Medical management
Final outcome	Survived, discharged	Survived, discharged	Survived, discharged

CKD = chronic kidney disease; DLP = dyslipidemia; DM = diabetes mellitus; ECMO = extracorporeal membrane oxygenation; HTN = hypertension; LCx = left circumflex artery; LV = left ventricle; LVEF = left ventricular ejection fraction; MCS = mechanical circulatory support; PAPI = pulmonary artery pulsatility index; PCI = percutaneous coronary intervention; RCA = right coronary artery; RV = right ventricle; SCAI = Society for Cardiovascular Angiography and Interventions.

Despite resuscitation and inotropic support, the patient deteriorated to Society for Cardiovascular Angiography and Interventions (SCAI) class D-E shock on day 2. Hemodynamics revealed a PAPI of 0.47 (PA pressure = 23/16 mm Hg, right atrial pressure = 15 mm Hg), confirming severe RV failure and prompting VA-ECMO/intra-aortic balloon pump initiation (Supplemental Table 1).

**Figure 2:**
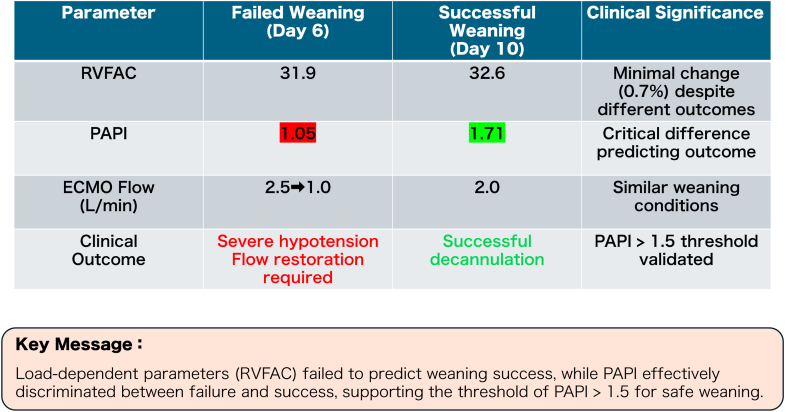
Comparison of Weaning Parameters in Case 1 Comparison of hemodynamic and echocardiographic parameters between failed weaning (day 6) and successful weaning (day 10) in case 1. Although right ventricular fractional area change (RVFAC), a load-dependent parameter, showed a minimal change (31.9% vs 32.6%), the pulmonary artery pulsatility index (PAPI) showed a significant improvement (1.05 vs 1.71), correctly predicting weaning success. This supports the utility of PAPI >1.5 as a weaning threshold. ECMO = extracorporeal membrane oxygenation.

Day 6 weaning was attempted, guided by an RVFAC of 31.9%. Reducing ECMO flow caused severe hypotension requiring flow restoration. The PAPI at this failed attempt was 1.05, below the threshold of 1.5. Retrospectively, the use of inhaled nitric oxide (iNO) during this initial weaning attempt might have facilitated successful liberation by reducing RV afterload because the patient was already on mechanical support, which would have prevented the LV decompensation risk. After optimization, the PAPI improved to 1.71 by day 9. Day 10 weaning succeeded with a PAPI of 1.71. Critically, RVFAC showed a minimal difference between failed (31.9%) and successful (32.6%) attempts, highlighting its unreliability as a stand-alone criterion (Figure 2, Video 1).

#### Key findings

Load-dependent parameters (RVFAC) cannot reliably predict weaning readiness. The PAPI threshold of 1.5 proved critical—weaning failed at 1.05 but succeeded at 1.71, supporting the potential value of PAPI in assessing hemodynamic competence during ECMO liberation.

### Case 2: ECMO with successful iNO during weaning

A 68-year-old man with diabetes and smoking history presented with 4-day fatigue culminating in severe chest pain. His blood pressure was 85/58 mm Hg and heart rate was 45 beats/min, with cold extremities. Arterial blood gas analysis showed metabolic acidosis (lactate level = 7.1 mmol/L, SCAI class C). Emergency angiography revealed 100% RCA in-stent restenosis (RCA #2), which was successfully treated with TIMI flow grade 3.

Twelve hours post-PCI, the patient deteriorated with recurrent acidosis, progressing to SCAI class D-E. Right heart catheterization confirmed severe RV failure with a PAPI of 0.64 (PA pressure = 18/7 mm Hg, CVP = 17 mm Hg), prompting VA-ECMO/intra-aortic balloon pump initiation.

Rapid improvement occurred, with day 3 PAPI increasing to 1.73. Day 4 comprehensive assessment at reduced ECMO flow (1.5 L/min) revealed PAPI = 2.00, s'/PASP = 0.47, and TAPSE/PASP = 0.68. During weaning, iNO (20 ppm) was administered for suspected residual pulmonary vascular resistance (PVR) elevation. Post-iNO hemodynamics showed that the PAPI increased to 2.5, whereas coupling ratios remained stable (s'/PASP = 0.47 → 0.44, TAPSE/PASP = 0.68 → 0.63) (Table 2). After successful clamp trial, decannulation occurred after 60 hours with robust postdecannulation parameters (PAPI = 2.5, TAPSE = 15.7 mm) (Video 2).

**Table 2:** Serial Hemodynamic and Echocardiographic Parameters With iNO

	Case 2: Pre-iNO (Day 4, ECMO 1.5 L)	Case 2: Post-iNO	Case 3: Pre-iNO (Day 2)	Case 3: Post-iNO
Invasive hemodynamics				
PAP (mm Hg)	20/10	25/10	42/14	32/25
CVP (mm Hg)	5	6	16	15
PCWP (mm Hg)	—	—	12-14	24
PAPI	2.00	2.50	1.75	0.87
Echocardiography				
TAPSE (mm)	13.6	15.7	—	15.4
s' (cm/s)	9.4	11.0	—	12.0
PASP (mm Hg)^a^	20	25	—	29.8
s'/PASP	0.47	0.44	0.46	0.40
TAPSE/PASP	0.68	0.63	0.58	0.52
RVFAC (%)	44.6	39.9	46	—
Clinical interpretation				
Response type	—	Beneficial	—	Harmful
Action taken	—	Continue then wean	—	STOP immediately

CVP = central venous pressure; ECMO = extracorporeal membrane oxygenation; iNO = inhaled nitric oxide; PAP = pulmonary artery pressure; PAPI = pulmonary artery pulsatility index; PASP = pulmonary artery systolic pressure; PCWP = pulmonary capillary wedge pressure; RVFAC = right ventricular fractional area change; s' = tricuspid annular systolic velocity; TAPSE = tricuspid annular plane systolic excursion; TRPG = tricuspid regurgitation pressure gradient.

a Doppler-derived estimation from TRPG when invasive measurement unavailable.

#### Key findings

Early decannulation (60 hours) was safely achieved when all parameters exceeded thresholds: PAPI = 2.00, s'/PASP = 0.47 (well above the 0.33 threshold), and TAPSE/PASP = 0.68 (exceeding the 0.41 threshold). Stability of coupling ratios during iNO therapy confirmed preserved intrinsic RV function despite pharmacologic intervention, demonstrating the value of comprehensive assessment in identifying patients ready for early liberation.

### Case 3: Conservative management with failed iNO trial

**Figure 3:**
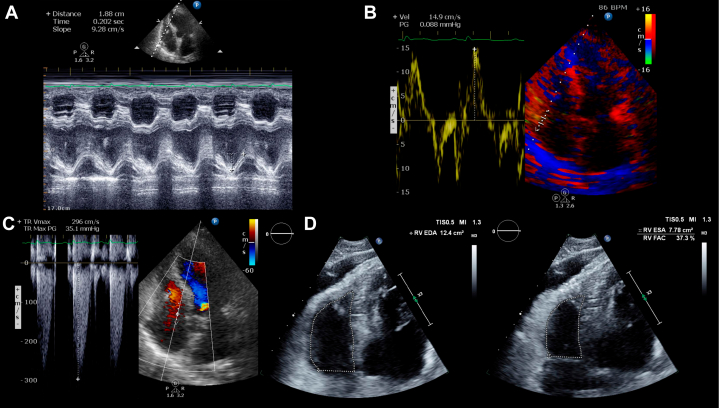
Echocardiographic Assessment Methodology Standardized echocardiographic measurements for right ventricular-pulmonary artery coupling assessment. (A) Tricuspid annular plane systolic excursion (TAPSE) by M-mode imaging. (B) Tricuspid annular systolic velocity (s’) by tissue Doppler imaging. (C) Tricuspid regurgitation pressure gradient (TRPG) for pulmonary artery systolic pressure estimation; coupling ratios (s’/PASP, TAPSE/PASP) are derived from these measurements. (D) Right ventricular fractional area change (RVFAC) by end-diastolic and end-systolic area tracings. Panels (A-C) obtained from Case 3 (acute phase) demonstrate coupling ratio assessment from a consistent hemodynamic state; Panel D from Case 2 (recovery phase) illustrates RVFAC methodology. TRPG = tricuspid regurgitation pressure gradient; other abbreviations as in Figure 1.

An 83-year-old woman with hypertension presented with cardiogenic shock due to dominant left circumflex artery stenosis (#11) supplying the RV (RCA hypoplasia). On norepinephrine, her blood pressure was 92/54 mm Hg with cold, clammy extremities. Electrocardiography showed complete atrioventricular block with ST-segment elevation; the lactate level was 5.2 mmol/L (SCAI class C). After successful PCI achieving TIMI flow grade 3, day 2 right heart catheterization yielded a PAPI of 1.05 (PA pressure = 35/17 mm Hg, CVP = 17 mm Hg), near the ECMO threshold. Baseline echocardiography revealed a left ventricular ejection fraction (LVEF) of 39.0% with posterior-inferior severe hypokinesis and a pulmonary capillary wedge pressure (PCWP) of 12 to 14 mm Hg, indicating limited LV reserve. RV assessment showed coupling ratios above minimal thresholds (s'/PASP = 0.46, TAPSE/PASP = 0.58) and preserved RVFAC (46%) (Figure 3). These parameters, combined with the absence of severe end-organ dysfunction, supported medical management over immediate ECMO.

**Figure 4:**
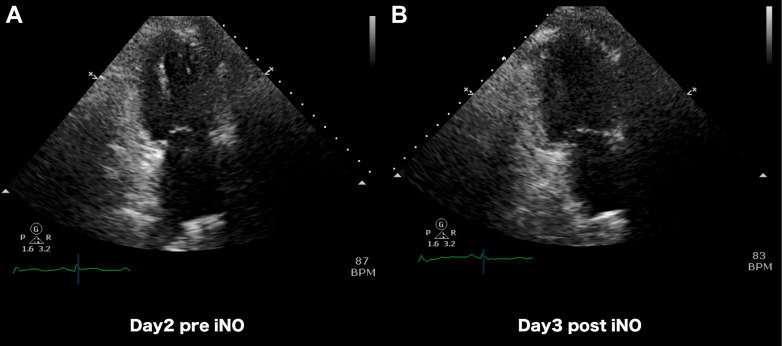
Paired Echocardiographic Images Demonstrating iNO-Induced Left Ventricular Volume Shift (Case 3) Apical 2-chamber views from case 3 at (A) day 2 baseline and (B) day 3 after inhaled nitric oxide initiation. Note the small, underfilled left ventricular (LV) cavity at baseline (A), reflecting reduced LV preload due to right ventricular (RV) dysfunction. After inhaled nitric oxide (iNO) administration (B), the LV cavity appears more filled, visualizing the mechanism whereby iNO-induced pulmonary vasodilation increased RV forward flow and LV preload. In this patient with limited LV reserve (left ventricular ejection fraction 39%), this acute volume shift resulted in LV decompensation (pulmonary capillary wedge pressure rose from 12-14 to 24 mm Hg).

On day 3, with rising PASP (42/14 mm Hg), iNO was initiated. Paradoxically, the PAPI dropped to 0.87 with PCWP increasing from 12-14 to 24 mm Hg, indicating LV decompensation from increased venous return (Figure 4). Coupling ratios also declined (s'/PASP = 0.46 → 0.40, TAPSE/PASP = 0.58 → 0.52) but less dramatically than PAPI, demonstrating that iNO failed to improve RV-PA coupling while paradoxically increasing venous return. iNO was immediately discontinued. Day 10 echocardiography during transient heart failure exacerbation provided additional insights. Despite preserved contractility metrics (TAPSE = 14.3 mm, s' = 15.1 cm/s, RVFAC = 48%), coupling ratios declined (s'/PASP = 0.43, TAPSE/PASP = 0.43), highlighting their sensitivity in detecting hemodynamic shifts from passive pulmonary pressure increase due to left heart failure (Video 3, Video 4, Video 5).

#### Key findings

Adequate coupling ratios (s'/PASP = 0.46, TAPSE/PASP = 0.58) provided decisive information for successful conservative management despite a borderline PAPI (1.05), demonstrating that coupling ratios can independently identify patients manageable without ECMO. The PAPI decline post-iNO (1.75 → 0.87) with a PCWP rise to 24 mm Hg revealed the critical importance of LV reserve capacity (baseline LVEF and PCWP assessment) for safe iNO use without mechanical support.

## Discussion

### Proposed framework and physiological integration

These 3 cases illustrate a potential integrated management framework that warrants prospective validation. The fundamental challenge is distinguishing true myocardial recovery from ECMO-induced artificial improvement. Ventricular-arterial coupling, defined as the relationship between ventricular contractility (end-systolic elastance, *E*_es_) and afterload (arterial elastance, *E*_a_), is central to RV performance.^3^^,^^9^ RVMI primarily disrupts this coupling through acute reduction in contractility, whereas secondary increases in PVR further compromise the relationship. VA-ECMO profoundly alters this physiology. Although RV unloading potentially limits infarct extension, loss of pulsatile flow reduces coronary perfusion pressure. In addition, increased LV afterload from arterial return exacerbates ventricular interdependence through septal shift, creating a complex hemodynamic milieu that confounds traditional assessment parameters.

### Applicability to biventricular infarction

This framework addresses RV-predominant shock; biventricular infarction requires modification. In such cases, PAPI interpretation becomes complex because concurrent LV dysfunction affects both PA pulse pressure and CVP. Coupling ratios may be confounded by secondary pulmonary hypertension from LV failure rather than intrinsic RV-PA uncoupling. The incidence of isolated RVMI requiring mechanical circulatory support remains poorly characterized, though cardiogenic shock develops in approximately 5% to 10% of RVMI cases.

### Literature-based thresholds

Our proposed thresholds integrate existing literature with our observations. Although multiple studies show that PAPI <1.0 predicts adverse outcomes in RV failure,^4^^,^^6^ case 3 (PAPI = 1.05) achieved successful conservative management with adequate coupling ratios (s'/PASP = 0.46, TAPSE/PASP = 0.58), suggesting that PAPI ≤1.0 should be interpreted alongside coupling parameters rather than as an absolute ECMO indication.

PAPI >1.5 represents a conservative approach for successful weaning, recognizing that ECMO liberation requires hemodynamic reserve.^5^^,^^6^ For RV-PA coupling ratios, we adopt thresholds validated by Park et al^8^ (s'/PASP >0.33, TAPSE/PASP >0.41).

### iNO therapy and LV reserve capacity

**Figure 5:**
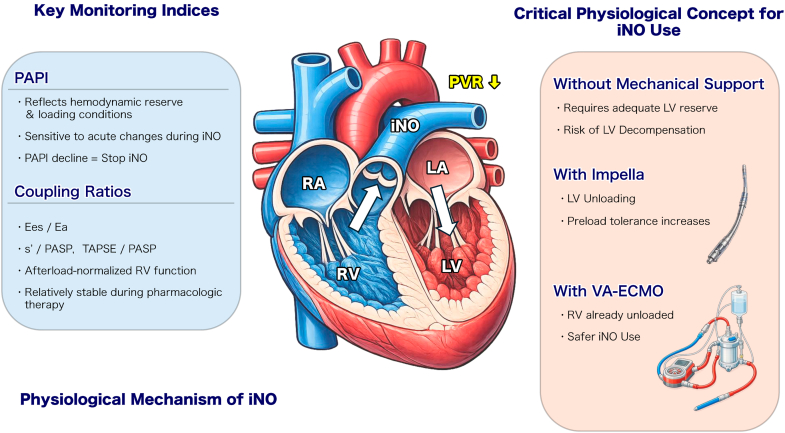
Physiological Mechanisms and Safety Criteria for iNO Therapy in Right Ventricular Myocardial Infarction Schematic illustration of the hemodynamic effects of inhaled nitric oxide (iNO) and safety considerations based on mechanical circulatory support status. Left panel: Key monitoring indices including the pulmonary artery pulsatility index (PAPI) for hemodynamic reserve assessment and coupling ratios (s'/PASP and TAPSE/PASP) for intrinsic right ventricular (RV) function evaluation. Center panel: Physiological mechanism of iNO—pulmonary vasodilation reduces RV afterload, increasing RV forward flow and left ventricular (LV) preload. Right panel: Safety criteria stratified by mechanical support status. Without mechanical support, iNO requires adequate LV reserve (left ventricular ejection fraction >40%-45%, pulmonary capillary wedge pressure <18 mm Hg) to tolerate increased preload; RV unloading with Impella or VA-ECMO permits safer iNO administration by managing the augmented venous return. Continuous monitoring of PAPI trend and coupling ratios guides therapy continuation or discontinuation. *E*_a_ = arterial elastance; *E*_es_ = end-systolic elastance; LA = left atrium; LVEF = left ventricular ejection fraction; PASP = pulmonary artery systolic pressure; PCWP = pulmonary capillary wedge pressure; PVR = pulmonary vascular resistance; RA = right atrium; s' = tricuspid annular systolic velocity; TAPSE = tricuspid annular plane systolic excursion; VA-ECMO = venoarterial extracorporeal membrane oxygenation.

iNO selectively dilates pulmonary vasculature, reducing PVR.^10^ Our experience demonstrates that iNO utility depends critically on LV reserve capacity. Case 2 demonstrated safe iNO use during ECMO weaning because ECMO provided RV unloading. Case 1 might have benefited from iNO during the failed weaning attempt because mechanical support would have prevented LV decompensation. Case 3 illustrated the critical importance of LV reserve for iNO without mechanical support. Despite an adequate baseline PAPI (1.75), iNO caused paradoxical deterioration. The mechanism is as follows: iNO reduces PVR → increases RV forward flow → increases LV preload. Without adequate LV reserve, LV decompensation occurs (PCWP rise to 24 mm Hg, PAPI decline to 0.87) (Figure 5). PAPI's sensitivity to acute hemodynamic changes during iNO serves as a real-time safety monitor. Coupling ratios showed relative stability during pharmacologic intervention, confirming their value for assessing intrinsic RV function.

For iNO without mechanical support, we recommend baseline assessment of LV reserve capacity including: 1) LVEF >40% to 45%; 2) baseline PCWP <18 mm Hg; and 3) absence of significant LV systolic dysfunction. These thresholds warrant validation but provide practical guidance for risk stratification.

### Relationship with SCAI shock classification

Our cases demonstrate how hemodynamic parameters complement clinical staging (Table 3).^7^ Cases 1 and 2, both progressing to SCAI class D-E, required ECMO despite different initial PAPI values (0.47 vs 0.64), suggesting that PAPI ≤1.0 may correspond to severe RV-predominant shock within SCAI class D-E. Case 3 (SCAI class C, PAPI = 1.05) succeeded with medical management, demonstrating that adequate coupling ratios may identify patients at the ECMO threshold who can be managed conservatively. These observations suggest that the echo-hemodynamic triad provides quantitative refinement of SCAI staging for RV-predominant shock, though validation in larger cohorts is essential.

**Table 3:** Algorithm Application to Case Management Decisions

	ECMO Weaning Decision: PAPI Threshold Met (≤1.0)	Decision	ECMO Weaning Decision: PAPI >1.5	ECMO Weaning Decision: s'/PASP >0.33 And/or TAPSE/PASP >0.41	Both Criteria Met	Outcome
Case 1	0.47 (met)	ECMO initiated	1.05	Day 4 values^a^: s'/PASP 0.32, TAPSE/PASP 0.63	No	Failed weaning (day 6)
			1.71	N/A^b^	Incomplete data^b^	Successful weaning (day 10)
Case 2	0.64 (met)	ECMO initiated	2.00	s'/PASP 0.47 (met), TAPSE/PASP 0.68 (met)	Yes	Successful weaning
Case 3	1.05 (not met)	Medical management	N/A	s'/PASP 0.46 (met), TAPSE/PASP 0.58 (met)	N/A	No ECMO needed

ECMO = extracorporeal membrane oxygenation; N/A = not available; PAPI = pulmonary artery pulsatility index; PASP = pulmonary artery systolic pressure; s' = tricuspid annular systolic velocity; TAPSE = tricuspid annular plane systolic excursion.

a Case 1 (day 6, first weaning attempt): Coupling ratios measured on day 4 (s'/PASP 0.32, TAPSE/PASP 0.63) were considered equivalent to day 6 values based on clinical stability.

b Case 1 (day 10): Despite incomplete coupling ratio data, successful weaning was achieved based on PAPI >1.5 and clinical stability, highlighting PAPI's critical role when comprehensive echocardiographic assessment is not feasible.

### Proposed clinical framework

Based on these observations and existing literature, we propose the following framework for future validation (Table 4):

• Initial Assessment Postrevascularization:
  ○ PAPI ≤1.0 with impaired coupling: May indicate need for VA-ECMO consideration.
  ○ PAPI >1.0 with adequate coupling (s'/PASP >0.33): Medical management may be feasible with close monitoring.
• ECMO Weaning Criteria:
  ○ PAPI >1.5 and s'/PASP >0.33 (primary predictor) and/or TAPSE/PASP >0.41.
  ○ Consider iNO for residual PVR elevation during weaning.
• iNO Therapy—Critical Prerequisites:
  ○ Without mechanical support: May require adequate LV reserve (baseline LVEF >40%-45%, PCWP <18 mm Hg, no significant LV dysfunction).
  ○ With ECMO: RV already unloaded, safer application.
• iNO Monitoring:
  ○ PAPI decline from baseline = immediate discontinuation.
  ○ Coupling ratios provide stable function assessment.

**Table 4:** Proposed Integrated Management Framework

Clinical Scenario	PAPI	Coupling Ratios (s'/PASP and/or TAPSE/PASP)	Recommended Action
Initial ECMO decision			
Severe RV failure	≤1.0	Any	Consider ECMO
Borderline RV failure	1.0-1.5	<0.33 and <0.41	Close monitoring, optimize medically
Adequate compensation	>1.5	s'/PASP >0.33 and/or TAPSE/PASP >0.41	Medical management
ECMO weaning trial			
Ready for trial	>1.5	s'/PASP >0.33 and/or TAPSE/PASP >0.41	Reduce flow, assess response
Not ready	<1.5	<0.33 and <0.41	Continue optimization
iNO therapy initiation			
Good candidate	Residual elevated PVR (with ECMO/Impella)	s'/PASP >0.33 and/or TAPSE/PASP >0.41	May initiate with close monitoring
Marginal candidate	<1.0 with adequate LV reserve	s'/PASP >0.33 and/or TAPSE/PASP >0.41	May consider if LV function adequate
Poor candidate	<1.5 and severe LV dysfunction (without support)	Any	Avoid; risk > benefit
iNO therapy monitoring			
Beneficial response	Stable or increased	Stable	Continue therapy
Harmful response	Decrease from baseline	Decreased	DISCONTINUE immediately

ECMO = extracorporeal membrane oxygenation; iNO = inhaled nitric oxide; LV = left ventricle; PAPI = pulmonary artery pulsatility index; PASP = pulmonary artery systolic pressure; PVR = pulmonary vascular resistance; s' = tricuspid annular systolic velocity; TAPSE = tricuspid annular plane systolic excursion.

### Study limitations

This study has important limitations inherent to the case series. First, with only 3 patients and no control group, statistical analysis is precluded, and causality cannot be established. The proposed framework is strictly hypothesis-generating. Second, selection bias is inherent—these cases represent successful management at our institution, potentially overestimating effectiveness. Third, generalizability is limited by single-center experience, heterogeneity of presentations, and variations in intervention timing. Fourth, the PAPI threshold is based on limited cases, and incomplete coupling ratio data restrict comprehensive validation. Fifth, PVR calculations were not performed during ECMO due to unreliable cardiac output measurement. Finally, the absence of standardized protocols reflects real-world practice but limits reproducibility. These observations are exploratory and require prospective validation with predefined protocols, larger cohorts, and control groups.

## Conclusions

Through analysis of 3 RVMI cases, we present preliminary observations for an integrated echo-hemodynamic framework requiring prospective validation. Key observations from these cases are as follows: 1) PAPI ≤1.0 with impaired coupling may suggest consideration for mechanical support; 2) successful weaning may require PAPI >1.5 and s'/PASP >0.33 (and/or TAPSE/PASP >0.41); 3) iNO appears to require adequate LV reserve without mechanical support; 4) PAPI decline during iNO may mandate immediate discontinuation; and 5) early consideration of iNO during ECMO weaning may facilitate liberation. These observations are hypothesis-generating and require validation in larger prospective studies.

**Visual Summary:**
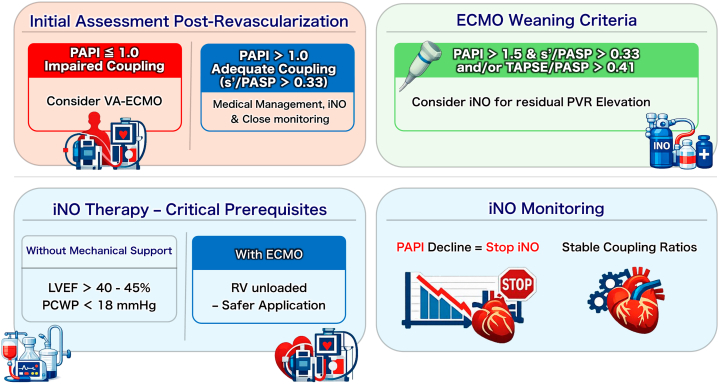
Proposed Clinical Framework for Echo-Hemodynamic Guided Management of Right Ventricular Myocardial Infarction Integrated decision-making algorithm for mechanical circulatory support in right ventricular (RV) myocardial infarction with cardiogenic shock. Upper left (initial assessment): Postrevascularization evaluation using a PAPI threshold of ≤1.0 to identify patients requiring VA-ECMO vs medical management. Upper right (ECMO weaning criteria): Dual-parameter approach requiring both PAPI >1.5 and adequate coupling ratios (s'/PASP >0.33 and/or TAPSE/PASP >0.41) before weaning attempts; inhaled nitric oxide (iNO) may facilitate RV recovery during mechanical support. Lower left (iNO therapy prerequisites): Safety criteria for iNO administration without mechanical support, requiring preserved left ventricular function (left ventricular ejection fraction >40%-45%) and normal filling pressures (pulmonary capillary wedge pressure <18 mm Hg). Lower right (iNO monitoring): Real-time assessment of therapeutic response; PAPI decline or deteriorating coupling ratios mandate immediate iNO discontinuation. PAPI = pulmonary artery pulsatility index; PASP = pulmonary artery systolic pressure; PVR = pulmonary vascular resistance; s' = tricuspid annular systolic velocity; TAPSE = tricuspid annular plane systolic excursion; VA-ECMO = venoarterial extracorporeal membrane oxygenation.

## Funding Support and Author Disclosures

The authors have reported that they have no relationships relevant to the contents of this paper to disclose.

## Appendix

Video 1 Case 1 Recovery Phase (Day 19) Apical 4-chamber view demonstrating recovered right ventricular function before discharge. Note: video recording during the acute phase was unavailable due to institutional archiving limitations.

Video 2 Case 2 During ECMO Support (Day 4) Right ventricular (RV)–focused apical 4-chamber view on day 4 during venoarterial-ECMO support. Preserved RV wall motion is visible despite mechanical unloading, corresponding to the coupling ratios (tricuspid annular systolic velocity/pulmonary artery systolic pressure = 0.47) that guided the successful weaning decision. ECMO = extracorporeal membrane oxygenation.

Video 3 Case 3 Baseline RV Assessment (Day 2) Apical 4-chamber view at admission. The right ventricle (RV) shows mild dilatation without severe enlargement. This visual finding, combined with preserved RV fractional area change (46%), supported the initial decision for conservative management.

Video 4 Case 3 Baseline LV Assessment (Day 2) Apical 2-chamber view at admission. The left ventricular (LV) cavity appears small and underfilled, reflecting reduced LV preload due to right ventricular dysfunction and ventricular interdependence.

Video 5 Case 3 Hemodynamic Shift Post-iNO (Day 3) Apical 2-chamber view after inhaled nitric oxide (iNO) initiation. Compared with day 2 (Video 4), the left ventricular (LV) cavity appears more filled and dilated. This visualizes the physiological mechanism where iNO reduced pulmonary vascular resistance and increased right ventricular forward flow, leading to increased LV preload. In this patient with limited reserve, this acute volume load resulted in LV decompensation (pulmonary capillary wedge pressure rose to 24 mm Hg).

Supplemental Table 1 Comprehensive Hemodynamic and Echocardiographic Data.
