# Supplementary material for: Integrated Echo-Hemodynamic Framework for VA-ECMO in Right Ventricular Infarction
Source: JACC Case Rep. 2026 Mar 25;31(18):107617. doi: 10.1016/j.jaccas.2026.107617 (PMC13154082; doi:10.1016/j.jaccas.2026.107617)
Supplement: Supplemental Table 1 — Comprehensive Hemodynamic and Echocardiographic Data. [file mmc6.docx]

**Supplementary Materials　Table . Comprehensive Hemodynamic and Echocardiographic Data**

**(Case 1)**

| **Parameter** | **Day 2** | **Day 4** | **Day 6** | **Day 7** | **Day 8** | **Day 9** | **Day 10** |
| --- | --- | --- | --- | --- | --- | --- | --- |
| **ECMO Flow (L/min)** | — | 3.0 | 2.5 to 1.0 | 2.5 | 2.5 | 2.5 | 2.0 to 0 |
| **TAPSE (mm)** | N/A | 15.8 | N/A | N/A | N/A | N/A | N/A |
| **s' (cm/s)** | N/A | 8.0 | N/A | N/A | N/A | N/A | N/A |
| **RVFAC (%)** | N/A | 33.1 | 31.9 | 42.4 | N/A | 24.2 | 32.6 |
| **PASP (mmHg)*** | 23 | 25 | N/A | N/A | 28 | 26 | 26 |
| **PADP (mmHg)*** | 16 | 16 | N/A | N/A | 16 | 14 | 14 |
| **CVP (mmHg)*** | 15 | 9 | N/A | N/A | 10 | 7 | 7 |
| **PAPI*** | 0.47 | 1.00 | 1.05 | N/A | 1.2 | 1.71 | 1.71 |
| **s'/PASP** | N/A | 0.32 | N/A | N/A | N/A | N/A | N/A |
| **TAPSE/PASP** | N/A | 0.63 | N/A | N/A | N/A | N/A | N/A |
| **Clinical Event** | ECMO initiated | — | Failed Weaning | — | — | — | Successful weaning |

**(Case 2)**

| **Parameter** | **Day 2** | **Day 3** | **Day 4** | **Day 4 (iNO)** |
| --- | --- | --- | --- | --- |
| **ECMO Flow (L/min)** | — | 1.96 | 1.5 | — |
| **TAPSE (mm)** | N/A | N/A | 13.6 | 15.7 |
| **s' (cm/s)** | N/A | N/A | 9.4 | 11.0 |
| **RVFAC (%)** | N/A | N/A | 44.6 | 39.9 |
| **PASP (mmHg)*** | 18 | 29 | 20 | 25 |
| **PADP (mmHg)*** | 7 | 10 | 10 | 10 |
| **CVP (mmHg)*** | 17 | 11 | 5 | 6 |
| **PAPI*** | 0.64 | 1.73 | 2.00 | 2.50 |
| **s'/PASP** | N/A | N/A | 0.47 | 0.44 |
| **TAPSE/PASP** | N/A | N/A | 0.68 | 0.63 |
| **Clinical Event** | ECMO initiated | — | — | Successful weaning |

*Invasive measurement via right heart catheterization

**(Case 3)**

| **Parameter** | **Day 2** | **Day 3** | **Day 3 (iNO)** | **Day 10** |
| --- | --- | --- | --- | --- |
| **ECMO Flow (L/min)** | — | — | — | — |
| **TAPSE (mm)** | 18.8 | N/A | 15.4 | 14.3 |
| **s' (cm/s)** | 14.9 | N/A | 12.0 | 15.1 |
| **RVFAC (%)** | 46 | N/A | N/A | 48 |
| **PASP (mmHg)*†** | 32, 35 | 42 | 29, 32 | 35 |
| **PADP (mmHg)*** | 17 | 14 | 25 | N/A |
| **CVP (mmHg)*** | 17 | 16 | 15 | 5 |
| **PAPI*** | 1.05 | 1.75 | 0.87 | N/A |
| **s'/PASP** | 0.46 | N/A | 0.40 | 0.43 |
| **TAPSE/PASP** | 0.58 | N/A | 0.52 | 0.43 |
| **Clinical Event** | Conservative Rx | — | iNO stopped | HF exacerbation |

*Invasive measurement via right heart catheterization †Doppler-derived/Invasive

**Abbreviations:** CVP: Central venous pressure; ECMO: Extracorporeal membrane oxygenation; HF: Heart failure; iNO: Inhaled nitric oxide; N/A: Not available; PADP: Pulmonary artery diastolic pressure; PAPI: Pulmonary artery pulsatility index; PASP: Pulmonary artery systolic pressure; RVFAC: Right ventricular fractional area change; Rx: Treatment; s': Tricuspid annular systolic velocity; TAPSE: Tricuspid annular plane systolic excursion.
